# Mechanistic Insight into the Enzymatic Inhibition of β-Amyrin against Mycobacterial Rv1636: In Silico and In Vitro Approaches

**DOI:** 10.3390/biology11081214

**Published:** 2022-08-12

**Authors:** Md Amjad Beg, Sadaf, Anas Shamsi, Sibasis Sahoo, Mohd Yousuf, Mohammad Zeeshan Najm, Yahya Ahmad Almutawif, Asimul Islam, Abdulaziz A. Aloliqi, Fareeda Athar

**Affiliations:** 1 Centre for Interdisciplinary Research in Basic Science, Jamia Millia Islamia, Jamia Nagar, New Delhi 110025, India; 2 Department of Biotechnology, Jamia Millia Islamia, Jamia Nagar, New Delhi 110025, India; 3 Membrane Protein Biology, International Centre for Genetic Engineering and Biotechnology (ICGEB), New Delhi 110067, India; 4 School of Biosciences, Apeejay Stya University, Gurugram 122103, India; 5 Department of Medical Laboratories Technology, College of Applied Medical Sciences, Taibah University, Madinah 42353, Saudi Arabia; 6 Department of Medical Biotechnology, College of Applied Medical Sciences, Qassim University, Buraydah 51542, Saudi Arabia

**Keywords:** Rv1636, β-Amyrin, molecular docking, molecular dynamics simulation, circular dichroism spectroscopy, isothermal titration calorimetry

## Abstract

**Simple Summary:**

Rv1636 is a mycobacterial universal stress protein whose expression level increases in different type of stress conditions. This protein promotes the growth of *Mycobacterium tuberculosis* in the host derived stress conditions generated during infection. Therefore in this manuscipt, we are trying to target Rv1636 using natural inhibitor. Targeting essential Mycobacterial protein using natural prodect was hypothesized to generate a molecule with low toxic effects and high inhibitory activity. It was found that Rv1636 contains ATPase activity and its ATPase activity gets disturbed by addition of β-Amyrin in the reaction. β-Amyrin was forund to interfere with the ATP binding site of Rv1636 which was confirmed by molecular docking anad dynamic studies. In addition to the ATPase activity, Rv1636 was also contain the cAMP binding capacity and also involved in balancing the cAMP levels inside cells. So, targeting Rv1636 using β-Amyrin disrupts its ATPase activity and cAMP regulatory activity and these conditions might make *Mycobacterium tuberculosis* more susceptible to the host derived stress conditions.

**Abstract:**

*Mycobacterium tuberculosis* has seen tremendous success as it has developed defenses to reside in host alveoli despite various host-related stress circumstances. Rv1636 is a universal stress protein contributing to mycobacterial survival in different host-derived stress conditions. Both ATP and cAMP can be bound with the Rv1636, and their binding actions are independent of one another. β-Amyrin, a triterpenoid compound, is abundant in medicinal plants and has many pharmacological properties and broad therapeutic potential. The current study uses biochemical, biophysical, and computational methods to define the binding of Rv1636 with β-Amyrin. A substantial interaction between β-Amyrin and Rv1636 was discovered by molecular docking studies, which helped decipher the critical residues involved in the binding process. VAL60 is a crucial residue found in the complexes of both Rv1636_β-Amyrin and Rv1636-ATP. Additionally, the Rv1636_β-Amyrin complex was shown to be stable by molecular dynamics simulation studies (MD), with minimal changes observed during the simulation. In silico observations were further complemented by in vitro assays. Successful cloning, expression, and purification of Rv1636 were accomplished using Ni-NTA affinity chromatography. The results of the ATPase activity assay indicated that Rv1636’s ATPase activity was inhibited in the presence of various β-Amyrin concentrations. Additionally, circular dichroism spectroscopy (CD) was used to examine modifications to Rv1636 secondary structure upon binding of β-Amyrin. Finally, isothermal titration calorimetry (ITC) advocated spontaneous binding of β-Amyrin with Rv1636 elucidating the thermodynamics of the Rv1636_β-Amyrin complex. Thus, the study establishes that β-Amyrin binds to Rv1636 with a significant affinity forming a stable complex and inhibiting its ATPase activity. The present study suggests that β-Amyrin might affect the functioning of Rv1636, which makes the bacterium vulnerable to different stress conditions.

## 1. Introduction

Tuberculosis is still a disastrous epidemic in many countries and has already claimed countless lives. *Mycobacterium tuberculosis* H_37_Rv (*M. tuberculosis*), the disease’s causative agent, can also enter the latent phase in the host cell, empowering its ability to evade the host immune system [1,2]. In the latent phase, the bacteria form and reside in a multinuclear set of immune cells called a granuloma. This structure provides *M. tuberculosis* with several strategic behaviors to escape from the immune system and delineate the infection in a containment site [3]. Numerous studies have been carried out and are still in the queue to define the factors responsible for granuloma formation [4]. Granuloma formation is known to be influenced by *M. tuberculosis* metabolic signaling cascades, virulence factors, secondary messengers, stress control machinery, and calcium regulation. In addition, *M. tuberculosis* has a remarkable stress regulation mechanism to stress out any unwanted discrepancy on the part of the host and utilize the nutrients for its existence [5,6]. Due to the bacterium’s unexpected and sophisticated character, it is vital to comprehend every facet of host–pathogen interaction as well as the various techniques that *M. tuberculosis* employs to rescue from the host immune system [7,8]. This investigation, along with the genes involved in the non-replicating/persisting phase, is still the primary objective of the research. Studies also predicted that *M. tuberculosis* experienced hypoxic conditions in the granuloma after infection of the host cell. This hypoxic environment was thought to be the main reason for the change in the active replicating state to the persistent non-replicating state of the bacteria [9,10,11].

Various previous studies generated the stress regulation system, and DosR/DosT/DosS regulon are expressed in response to hypoxia and nitric oxide (NO) stress and involve the differential expression of around 48 genes termed as DosR regulon [12,13,14,15]. Later, it was discovered that the DosR regulon controls the carbon monoxide (CO) stress in macrophages; therefore, it is believed to be an effective adaptation strategy in response to hypoxia, NO, and CO circumstances. The 48 differentially expressed genes contain six presumably conserved proteins known as Universal Stress Proteins (USP). Identification of these hypothetical proteins led to the discovery of genes responsible for augmenting the bacterium’s ability to endure under stressful conditions [16].

The USP domain comprises ~160 amino acids. USPs are promising therapeutic targets since they are extensively distributed among several organisms, including bacteria, plants, Archaea, and fungi, but they are nebulous in people [17]. Only Mycoplasma species lack USP, but all other archaebacteria and eubacteria have USP. The domain name first originated from *Escherichia coli* UspA, which was upregulated in response to various stresses [18,19,20,21,22]. *Pseudomonas aeruginosa* (*P. aeruginosa*) USPs take a role in bacterial endurance in the astute microbe under an anaerobic fixed site, which is thought to resemble those found in the cystic fibrosis lung colonized by *P. aeruginosa* [23,24]. A UspA freak in *Salmonella typhimurium*, an enteric bacterium, showed diminished endurance after supplement starvation and increased affectability to oxidative stress, and it makes a significant commitment to virulence. The structure analysis showed that the USP structure falls into two major groups: one binds with ATP and the other is non-ATP bound USP [25]. The formation of two major groups established the classification based on structure information of *Methanococcus jannaschii*, whose crystal structure MJ0577 bound to ATP, and structure information of *Haemophilus influenzae* had no clues for binding with ATP. The ATP binds to the putative ATP binding site having a G2 × G9 × GS motif [26].

There are ten USPs in *M. tuberculosis*, which are elevated in macrophages, indicating that they may be crucial for bacterial survival and persistence. These USPs are implicated in a variety of stressors on M. tuberculosis, including low pH, NO, UV light, antibiotic stress (such as mitomycin C), food shortage, etc. Similar to all other ATP-binding USPs, Rv1636 has a conserved aspartate (D) in the DGS motif in the USPA domain and a G2 × G9 × GS ATP binding site motif. Additionally, Rv1636 was identified as the sole USP homolog in *Mycobacterium leprae* (M. leprae), the causative agent of leprosy in humans [27,28]. Rv1636, now known as *M tuberculosis* sinking machinery, was later found to have a stronger affinity for binding to cAMP than ATP. M. leprae contains only one USP (Rv1636), and almost 89 percent of the amino acid sequences of M. leprae and M. tuberculosis are identical. Rv1636 has similar ATP binding capabilities to known USPs, but its cAMP binding is distinctive because it targets this gene and blocks cAMP signaling [29]. cAMP is the universal secondary messenger essential for survival and has been linked to numerous metabolic activities. The downregulation of effector proteins mediates the action of cAMP [30,31]. These proteins contain a cyclic nucleotide-binding domain (CNB domain), fused with the effector domain to regulate several processes such as catalytic activity, the transport of ions and small molecules, or binding DNA [32]. Furthermore, *M. tuberculosis* also effectively secreted cAMP, which perhaps helps modulate the host immune system in case of *M. tuberculosis* infection and establishes caseous granuloma [33,34]. Due to these facts, Rv1636 is a crucial and fascinating gene to research for developing and discovering innovative mechanisms to combat *M. tuberculosis* and reduce the TB burden.

With the present manuscript, we are attempting to identify the compound that will target the Rv1636 and obstruct an essential USP’s functioning through in silico, biochemical, and biophysical characterization. The article extends another study on the different phytoconstituents of three plants: *Achyranthes aspera*, *Calotropis gigantea*, and *Calotropis procera*. According to phytochemical research, extracts of these plants are a good source of natural flavonoids and polyphenols. The ethyl acetate fragmentation of *A. aspera aerial* and *C. gigantea* floral ash efficiently combats *M. tuberculosis* H_37_Rv ATCC 27,294 strains with a MIC value of 64 g/mL. The results of the GC-MS analysis of both plant fractions were used to create a list of compounds. Determining the target protein is a different investigation window; to accomplish this, two categories were shortlisted: available PDB dataset proteins and proteins categorized in virulence, detoxification, and adaptation categories. BpoC, RipA, MazF4, RipD, TB15.3, VapC15, VapC20, VapC21, TB31.7, and MazF9 were among the ten proteins available in both categories. Further molecular docking showed that β-Amyrin from the GC-MS resulted in the compound interacting with most of the proteins, and its highest binding affinity was with Rv1636 protein.

## 2. Materials and Methods

### 2.1. Sequence-Based Prediction of Rv1636 Protein: In Silico Studies

#### 2.1.1. Retrieve Rv1636 Protein Sequence

The Mycobrowser, a Mycobacterial searching browser, was used to obtain the sequence of Universal stress protein Rv1636 from *M. tuberculosis*. This server is an online database with an extensive library of infectious and non-infectious strains of mycobacterial proteins for extensive genomic and proteomic investigation [35,36].

#### 2.1.2. Physiochemical Parameters

The ProtParam server examined the identification of physical and chemical properties. ProtParam tool is a server that calculates the theoretical parameters, including molecular weight, amino acids, pI, instability index, and so on [37].

#### 2.1.3. Prediction of the Protein Localization

The mycobacterial protein localization is essential in developing a new therapeutic target for drug discovery. Because there was no information on the Rv1636 protein localization, the application of different in silico approaches by many servers such as TBpred, CELLO2GO, LocTree3, and PSORTb predicted the localization. In addition, multiple prediction algorithms, such as SVM, HMM, BLAST, and PSI-BLAST, were used to indicate the localization of Rv1636 protein based on the score [38,39,40,41].

#### 2.1.4. Secondary (2D) Structure Prediction

The secondary structure of Rv1636 protein sequences was predicted by various web servers such as SOPMA, PSIPRED, Jpred4, and PredictProtein. These online servers are accurate and straightforward prediction programs for identifying different forms of a secondary structure feature, such as alpha-helix, extended strands, or random coil area that contains the primary element of secondary structure determination [42,43,44,45].

#### 2.1.5. Phylogenetic Studies of Rv1636

The Rv1636 protein sequence was searched for significant alignment using BLASTp suit in the phylogeny reconstruction map, and a neighbor-joining tree was constructed using MEGA11. Bootstrap values are based on 1000 iterations for testing node significance. The Poisson model was used to calculate protein distances and uniform rates. Three threads are used to accomplish the gap opening penalty or missing data treatment via Pairwise deletion [46,47].

#### 2.1.6. Interaction Analysis (PPI and PCI)

In this interaction study, the PPI stands for protein–protein and PCI protein–compounds interaction. A protein–protein interaction network of Rv1636 was established using the STRING v11.5 (https://string-db.org/) and STITCH 5.0 (http://stitch.embl.de/) database was (accessed on 07 July 2021), whereas the selected cutoff score was 0.5–0.9 that showed the interaction with high confidence. The studied interactions were further analyzed by cutoff score results shown separately for each data. Scores of 0.4 to 0.7 showed medium interaction; a score > 0.7 showed high interaction with a significant statistical threshold of less than 0.05 [48,49,50].

### 2.2. Structure-Based Prediction of Rv1636 Protein: In Silico Studies

#### 2.2.1. Molecular Docking

The molecular docking studies were performed by InstaDock software, which demonstrates the presence of subatomic, which is used to identify the binding pattern of the protein-ligand. Further, the three-dimensional (3D) crystal structure of target protein Rv1636 (PDB ID: 1TQ8) was retrieved from RCSB PDB and refined before performing molecular docking [51,52]. Surprisingly, when visualizing the crystal structure 1TQ8, some residue positions are not in all the chains’ electron density maps. Our other studies have shown that Rv1636 is a potential virulent protein that overexpresses host-derived stress conditions in response to mycobacterial infection. An in vitro experiment revealed that Rv1636 has a substantial binding affinity for ATP and cAMP [53,54]. As a result, we performed molecular docking of -Amyrin with Rv1636 using these two compounds as a control. The ligands used in this study were obtained from the PubChem online resource database [55]. During receptor preparation, water atoms and co-crystallized ligands HETATM were removed from the PDB file. After adding more polar hydrogen bonds, the PDB file was converted to pdbqt format. The best-fitting conformation regarding ligand–receptor complex binding energies was determined while preserving the receptor as a site-specific binding, and the preferred ligand is flexible. This molecular docking research was performed using InstaDock software, and the results were reviewed using the log files, which are automated and generated in the result folder [56,57].

#### 2.2.2. Receptor–Ligand Interaction Profile

The receptor–ligand complex was created using Discovery Studio (BIOVIA). This software used interaction studies of dock conformations to understand better the ligand-binding amino acid residues with different bond formations [58,59].

#### 2.2.3. Pharmacokinetics Profile

The ligands with the highest binding energies were chosen in molecular docking to determine the drug ability assessment further using ADMETlab2.0 and the pkCSM website [60,61]. Commercially available internet web servers projected the pharmacokinetic profile of the selected natural chemical. Furthermore, computational approaches were used to identify compound toxicity, which is faster than finding dangerous levels in animals and can reduce the number of animal testing.

#### 2.2.4. Pharmacological Profile

In this context, predicting the pharmacologic target of the selected natural compound using PASS online (http://way2drug.com/PassOnline/, accessed on 23 July 2021) was also used to predict the biological activity spectrum of the chosen compound [62].

#### 2.2.5. Molecular Dynamics Simulation

All MD simulations were carried out with the Schrodinger Desmond tool to obtain insight into the binding stability of Rv1636: native and docked complex of beta-amyrin, cAMP, and ATP. Before MD simulations, both native and docked complex structures were minimized using the protein preparation wizard of Schrodinger, where the hydrogen bond network was optimized at pH 7.4, and final restrained minimization was performed using the OPLS3e force field.

Further minimized structures were incorporated into the workspace using a system builder module, an orthorhombic box solvated with a TIP3P water model, and 0.15 M NaCl counter ions. A series of energy minimization and short MD simulations relaxed all prepared systems before the MD simulation. Finally, the MD simulations were subjected to a 100 ns time for the individual approach and saved the coordinates at an interval of 50 ps at 300 K temperature with 1.0325 bar pressure [63].

The simulation results were further analyzed using the Simulation Event Analysis module in DESMOND, i.e., Root Mean Square Deviation (RMSD) and Root Mean Square Fluctuation (RMSF).

### 2.3. Determination of Protein Expression and Purification of Rv1636 Protein: In Vitro Studies

#### 2.3.1. Cloning of Rv1636 in pET28a Vector

The Rv1636 gene was amplified using the polymerase chain reaction method, and the genomic DNA of *M. tuberculosis* H37Rv served as a template. The sense and antisense strands of the reaction were primed with 5′ < GAGAATTCATGAGCGCCTATAAGACCGTGG > 3′ and 5′ < AACTCGAGCTAGGTGGTGTGCACGATCAGC > 3′, respectively. Restrictions sites for EcoRI and XhoI were present in FP and RP, respectively. Program: initial denaturation at 95 °C for 4 min, subsequent denaturation at 94 °C for 1 min, followed by annealing at 60 °C for 1 min, and extension at 72 °C for 1.30 min. Then, 30 cycles of this therapy should be completed with a final extension at 72 °C for 7 min. EcoRI and XhoI cut the amplified Rv1636 and the pET-28a vector to create compatible sticky ends. The overnight ligation was carried out at 16 °C in the water bath. The ligation reaction was heat-inactivated at 65 °C and transferred into DH5α cells. The plasmid was extracted from the screened colonies, and the expression construct was verified using restriction digestion and colony PCR, both of which showed a band of a similar size. Subsequently, the plasmid was transferred into a chemically competent BL-21 (DE3) strain [64].

#### 2.3.2. Protein Expression and Purification

Transformants from the BL-21 (DE3) strain were injected with 50 µg/mL kanamycin in Luria Bertani broth media and agitated overnight in an incubator shaker at 220 rpm at 37 °C. Isopropyl—thiogalactoside (IPTG) was added to the medium when the OD600 pointed at 0.7–0.9, and the liquid suspension was further incubated for 3–4 h to induce the protein. First, the cells were pelleted down using centrifugation for 30 min/10,000 rpm at 4 °C. Then, the pelleted cells were resuspended in a resuspension buffer (50 mM Tris-Cl pH8.0, 300 mM NaCl, 10% Glycerol, 1 mM PMSF) lysed by sonicating the cells for 7 min. Next, the lysed cells were centrifuged for 30 min to remove cell debris. After 3 h of mixing with Ni-NTA resin, the supernatant was washed with washing buffer (50 mM Tris-Cl pH8.0, 300 mM NaCl, 10% Glycerol, 20 mM imidazole). The protein was eluted in the elution buffer, which is the same as washing with the addition of 0.2 M imidazole [65].

### 2.4. Biochemical and Biophysical Characterization of Rv1636 Protein: In Vitro Studies

#### 2.4.1. Biochemical Characterization of Rv1636 to Determine Its ATPase Activity

The malachite green phosphatase assay was used to assess the enzymatic activity of Rv1636, ensuing the methodology of (Veldhoven et al., 1987). The enzymatic reaction is monitored for the presence of a stable phosphomolybdate complex. The reactions were run in TMD buffer (50 mM Tris pH 8.0, 10 mM MgCl_2_, and 1 mM DTT), with the ultimate basal rate determined by the quantity of phosphate released in the presence of Rv1636. A separate reaction without Rv1636 conducts the detection of any non-enzymatic ATP hydrolysis throughout the reaction. This value was deducted from the Pi released due to enzymatic ATP hydrolysis. The reaction was carried out in a TMD buffer with a total volume of 50 µL and incubated in an MCT for one hour. After 1 h, the reaction was made up of 550 µL of autoclaved MQ water and further supplemented by 200 µL each of chemical A (1.75% (*w*/*v*) ammonium heptamolybdate 4H_2_O in 6.3 N H_2_SO_4_ and reagent C (0.035% malachite green and 0.35% PVA in water) to make a total reaction volume of 1 mL [66]. For 1 h, the reaction was kept at room temperature, followed by incubation to stabilize the phosphate and molybdate complex, followed by absorbance measurement at 610 nm. The procedure used a concentration of 0–80 µM ATP and calculated the kinetic characteristics by measuring the preliminary rate of nucleotide hydrolysis with 5 µM Rv1636.

Lineweaver Burk equations were used to determine the kinetic parameters. The y and x-intercepts approximate the reciprocal of *V_max_* and the negative reciprocal of *K*_m_ (Michelis Menton constant). Next, the *K*_m_ and *V*_max_ were calculated using a regression line equation to link the numbers. Finally, *V*_max_ was divided by total enzyme concentration to determine *K*_cat_ [63,67].

#### 2.4.2. Circular Dichroism (CD) to Determine the Secondary Structure of Rv1636

Using far UV-Vis CD, the secondary structure of Rv1636 and Rv1636 in association with amyrin were analyzed. All experiments were conducted using a cuvette with a 1 mm route length at 25 °C. First, a buffer containing 50 mM Tris pH 8.0 and 300 mM NaCl was combined with 5 M Rv1636 in a reaction volume of 300 L. The reaction was then carried out by raising the concentration of amyrin from 10 M to 10 M to 400 M after the ligand had been produced in the same buffer. Two different wavelengths—200 and 260 nanometers—were chosen. Both the native Rv1636 spectrum and the spectrum following the addition of the ligand were recorded and quantified. The built-in Jasco software examined the spectra, and the 20 protein structural patterns in combination with ligands were quantified with the BeStSel server [68,69].

#### 2.4.3. Isothermal Calorimetry (ITC) to Determine the Thermodynamic Properties of the Interaction of Rv1636 and β Amyrin

Isothermal calorimetry is a method for calculating the thermodynamic parameters of a two-molecule interaction. Binding affinity, enthalpy, and entropy are characteristics that can be calculated. The heat emitted or absorbed during a reaction processed in the form of ligand titration in protein is recorded, and the parameters were determined. ITC experiment was performed on MicroCal VP-ITC (Northampton, MA, USA). The solutions were degassed in a thermovac for 30 min at 25 °C before loading to remove bubbles. The sample cell is filled with the 2 mL of degassed 20 μM Rv1636 solution, and the reference cell is filled with the corresponding buffer. β Amyrin (500 µM) was loaded in the rotator syringe. The reference power was fixed at 16 μcal/s. The spacing amid two consecutive injections was 180 s and the time duration of all injections was kept at 20 s. The final figure was obtained using the MicroCal Origin 8.0, and the data were plotted using one model binding site [70,71].

## 3. Results

### 3.1. Physiochemical Parameters

The Rv1636 protein’s amino acid sequence was obtained from the Mycobrowser database prior to the physiochemical evaluation. The physicochemical properties for these proteins were calculated using the Protparam web service, as mentioned in Table 1. Physiochemical parameter of the proteins calculated the isoelectric point (pI) of Rv1636 is less than 7, which means it might be acidic. The instability index value determined that it is a stable protein, probably stable in the test tube. This protein’s calculated aliphatic index indicated that it was thermo-stable over a temperature range. Rv1636 was classified as polar based on the GRAVY (grand average hydropathy) value.

### 3.2. Subcellular Localization

Knowing where a protein is situated is critical to predicting its function. All different algorithm-based servers projected the Rv1636 protein in the cytoplasmic area, as shown in Table 2.

### 3.3. Secondary Structure Prediction

Various online servers made the two-dimensional (2D) structure prediction of the Rv1636 protein. All of these servers predicted that this protein is rich in the alpha helix region. SOPMA predicted 67%, artificial NNML predicted 65%, JNet algorithm predicted 54%, and the deep learning method showed 69% alpha-helix region. As shown in Table 3, all of these servers used the default settings to analyze the 2D structure of the Rv1636 protein and concluded that it had more alpha helix region.

### 3.4. Phylogenetic Analysis

**Figure 1:**
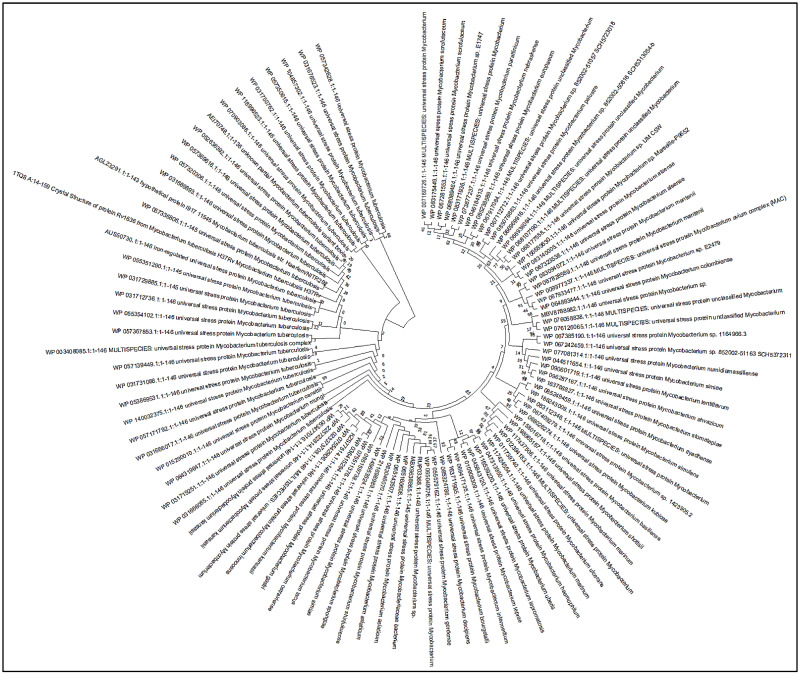
Phylogenetic tree depicting the relationships between *Rv1636* protein and *M. tuberculosis* species.

Phylogenetic exploration provides a detailed understanding of similar interest protein sequences which evolve because of genetic changes. Using BLASTp, I have searched similar sequences of Rv1636 in different strains and aligned them among the interspecies of mycobacteria in the Fasta format file. Mega11 phylogenetic analysis software constructs the tree with an algorithm neighbor-joining method found Rv1636 has a similar sequence length, retains the unique domains (G2XG9XGS), and has little amino acid change in length between different mycobacterial species. In addition, the path lengths of the homologs in distinct mycobacterial species were defined for each interaction, as illustrated in Figure 1.

### 3.5. PPI Interaction Studies of Rv1636

The PPI (protein–protein interaction) network analysis was performed using the STRING v11.0 database, and the result showed that the Rv1636 interacted with the TB16.3, uspA, TB31.7, Rv1360, TB9.4, TB18.6, mpt63, kdpD, ssb, and ufaA1. In this interaction study, the predicted score is (0.577 to 0.864). These interaction studies showed that there is experimental determination between protein is not present, rest all the interaction having text mining score is mentioned which is shown in Table 4.

Accordingly, the top score of interactions showed TB16.3 and uspA having the best interaction from the text mining approach. TB 16.3 is a conserved mycobacterial protein that plays a vital function in triggering the NF-κB signaling pathway, which is essential in host protective immunity, albeit the exact mechanism is unknown. Rv2316 (uspA) is an integral membrane protein involved in the active transport of sugar across the membrane. This protein oversees transporting sugar across the membrane (import).

**Figure 2:**
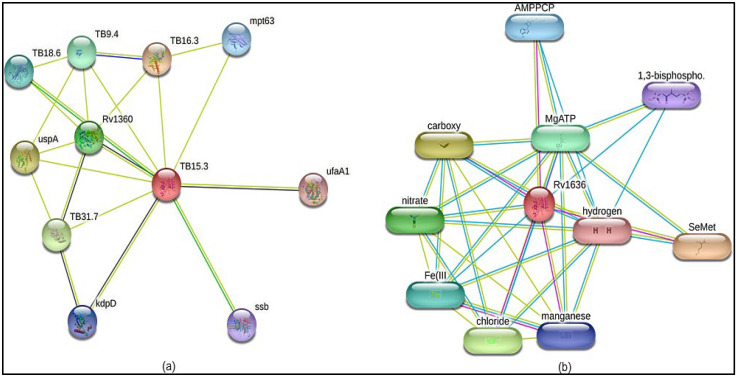
The interaction studies of Rv1636 protein. (**a**) Protein–protein interaction network and (**b**) protein–chemical interaction network.

The protein–chemical interaction using the STITCH database shows the Rv1636 chemically interacted with the SeMet, carboxy, chloride, nitrate, MgATP, Fe, AMPPCP, manganese, 1,3 bisphosphate, and hydrogen. In addition, previous in vitro studies proved that the Rv1636 highly interacted with cAMP and ATP. In Rv1636, the protein–protein and protein–chemical interaction studies are also illustrated in Figure 2.

### 3.6. Molecular Docking

The molecular docking analysis was performed using InstaDock v1.0 software; it includes the fundamental orientations between the receptor and the ligand (ATP, cAMP, and Beta-amyrin). Our molecular docking studies consist of the two known binders of Rv1636 cAMP and ATP for validating our docking protocol. The docked pose of ATP and cAMP has a docking score of −7.2 and −7.4 kcal/mol. The β-Amyrin (tri-terpenoid), a natural compound from the GC-MS library of (A. aspera and C. gigantea plant) has a docking score of −10.6 kcal/mol, which indicate that the binding affinity of this natural compound is likely to be stronger than the native ligand (ATP & cAMP) (Table 5). The highly negative docking score (binding free energy kcal/mol) was strong evidence to indicate that their complex binding is steady.

#### 3.6.1. Receptor–Ligand Interaction Profile

**Figure 3:**
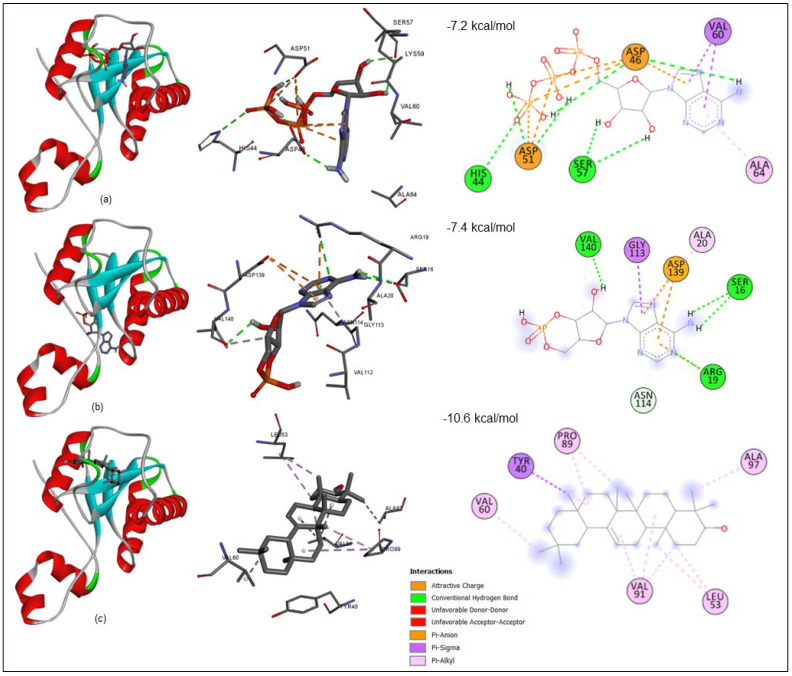
Docking interaction studies of 2-D structural representation of protein–ligand complexes. (**a**) Rv1636 with ATP; (**b**) Rv1636 with cAMP; (**c**) Rv1636 with β-Amyrin having pi-Alkyl bonds (purple) and Hydrogen bond (green) with their interacted residues.

Using PyMOL and the Discovery Studio (BIOVIA) visualizer, structural characterization of the receptor–ligand complex in various conformational states was performed. To identify protein–chemical interactions in a binding pocket of the protein, docking analysis of Rv1636 with ATP, cAMP, and -Amyrin was conducted. The findings revealed that all interactions predominate in the active pocket, as illustrated in Figure 3.

The comprehensive interactions between the compounds and the Rv1636 binding pocket were explored to investigate these interactions between β-Amyrin and control ligands (ATP and cAMP). A closer view at the binding site in Rv1636 suggests that different types of interactions between Rv1636 with ATP (HIS44, ASP46, ASP51, SER57, VAL60, and ALA64), R1636 with cAMP (SER16, ARG19, ALA20, GLY113, ASN114, ASP139, and VAL140) are hydrogen, Pi-anion, Pi-sigma, and Pi-alkyl bond but Rv1636 with β-Amyrin (TYR40, LEU53, VAL60, PRO83, VAL91, and ALA97) showed Pi-Alkyl and sigma bonds. In addition, molecular dynamics simulation analysis on prospective in vitro hits might provide valuable insights into chemical optimization.

#### 3.6.2. Pharmacokinetics Profile

The Admetlab2.0 and pkCSM server determined the pharmacokinetics profile study of beta amyrin, revealing the significant characteristic of drug action in a curative manner. We have studied the β-Amyrin has a set of good pharmacokinetic profiles such as Lipinski rule accepted, no PAINS pattern found. There is no thiol, chelating, and undesirable reactivity. The ADMET properties of beta-amyrin were also examined to ensure no potentially harmful patterns in their molecular structures. The complete results are given in Table 6.

#### 3.6.3. Pharmacological Profile

A machine learning approach based on structure-activity links can be applied in PASS online analysis estimation. The reference substance β-Amyrin showed a wide variety of biological activity potential in this example. Insulin promoter, Caspase 3 stimulant, Transcription factor stimulant, Mucomembranous protector, Hepatoprotectant, Apoptosis agonist, Antineoplastic, Oxidoreductase inhibitor, Membrane integrity antagonist, and Chemopreventive potential have all been found in β-Amyrin, with Pa (probability to be active) > Pi (probability to be inactive) and Pa values ranging from 0.977 to 0.903 as shown in (see Table 7).

#### 3.6.4. Molecular Dynamics Simulation

**Figure 4:**
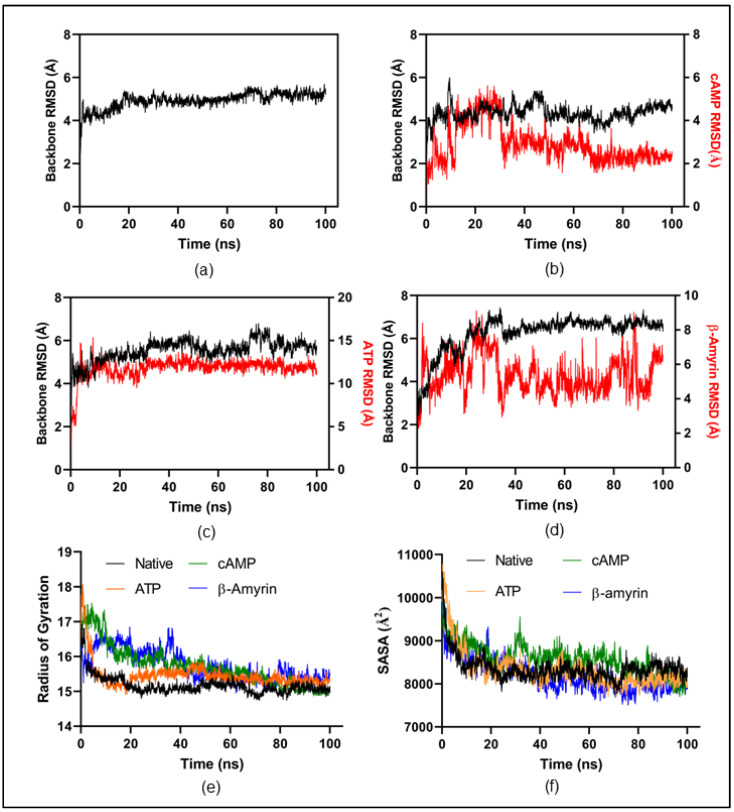
Molecular dynamics simulation of modeled native and ligand docked structure of Rv1636. (**a**) Native-Rv1636 backbone RMSD plotted against the time (ns). RMSD of docked complex (**b**) cAMP, (**c**) ATP, and (**d**) β-Amyrin. (**e**) The compactness of the whole Rv1636 structure is accessed through the radius of gyration. (**f**) Solvent accessible surface area throughout 100 ns MD simulation.

All-atomic MD simulations were carried out for four systems, including native-Rv1636 and three docked complexes of Rv1636: cAMP, ATP, and β-Amyrin, to investigate the stability and conformational changes following the binding of cAMP, ATP, and β-Amyrin in the binding pocket of Rv1636. The chemical structures of these following compounds are mention in Figure S1. The dynamic profile of Rv1636 was explored by the Root mean square deviation (RMSD) of the MD simulation trajectories of each system. The RMSD plots (Figure 4) have inferences that all three docked ligand complex systems obtained an equilibrium state after 40 ns and remained stable until the end of the MD simulation. The RMSD plot of the ligands demonstrated that cAMP has low RMSD values compared to ATP and β-Amyrin. Further ligand RMSD with respect to protein backbone was investigated for each ligand; RMSD of cAMP, ATP, and β-Amyrin was 2.9 ± 0.8 Å, 12.0 ± 1.2 Å, and 5.4 ± 1.1 Å, respectively, in Figure 4a–d.

The radius of gyration (*R*_g_) is a significant indicator in MD simulation, which is utilized to access the structural compactness of Rv1636 native and the other three docked ligands. Time-dependent plots of the radius of gyration for the native and three complexes are shown in Figure 4e. A similar pattern of compactness was observed in all simulations. In contrast, in the presence of β-Amyrin and cAMP, *R*_g_ was reduced from the initial state (~16–17 Å) to the end of the simulation, where *R*_g_ was recorded at 14.9–15.4 Å, implying more compactness to structure. Similarly, we compared the total change in solvent accessibility surface area (SASA) in each simulation depicted in Figure 4f. β-Amyrin binding significantly affects the solvent-exposed surface of Rv1636, which corroborates with the radius of gyration data. SASA values were found to be reduced throughout 100 ns MD simulation for the β-Amyrin (8200 ± 364 Å2) in comparison to other systems, i.e., native (8345 ± 297 Å2), ATP (8327 ± 430 Å2), and for cAMP (8576 ± 348 Å2).

**Figure 5:**
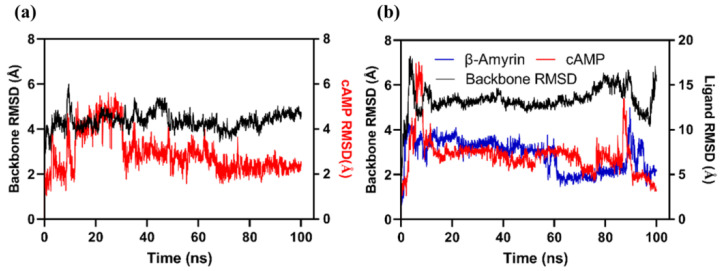
Molecular dynamics simulation of Rv1636-cAMP complex with and without β-Amyrin. (**a**) Evolution over time of the RMSD values of Rv1636-cAMP standalone complex. (**b**) RMSD values of the Rv1636 complex with cAMP and β-Amyrin.

Additionally, we also performed an all-atomic simulation for Rv1636 in a complex with cAMP to further decipher the binding mechanism. We carried out an independent simulation wherein β-Amyrin was crafted onto the Rv1636-cAMP complex structure (Figure 5). The RMSD analysis showed a higher backbone movement in the protein backbone where both cAMP and β-Amyrin ligands were present (Figure 4b). Whereas in the Rv1636 cAMP complex, the backbone was stabilized after an initial period of MD simulation. cAMP binding stability is also disrupted in the presence of β-Amyrin, in comparison to Rv1636-cAMP standalone complex.

### 3.7. Purification of Rv1636

**Figure 6:**
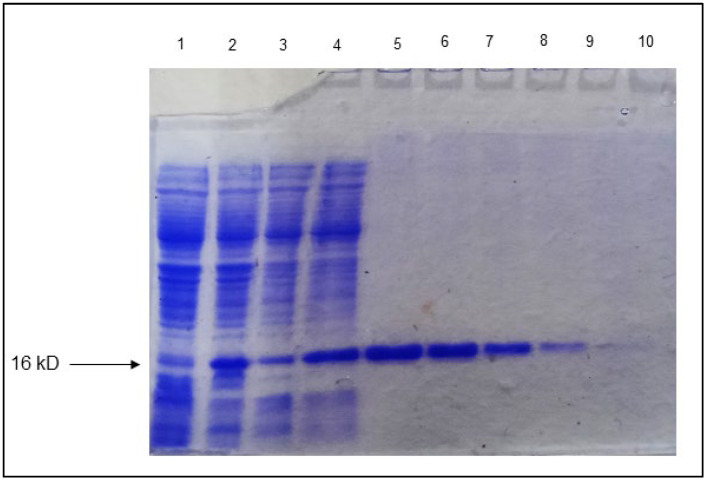
**Protein purification**: Protein purification of Rv1636: Lane 1 and 2: uninduced Rv1636 and induced Rv1636, Lane 3: wash 1, Lane 4: wash 2, Lane 5–10: Purified Rv1636 fractions 1, 2, 3, 5, and 7.

Rv1636 was successfully cloned into pET-28a+ using EcoRI and XhoI, and the protein was purified from BL-21 (DE3) strain constituted with N terminal His tag. The protein expression was optimized at 37 °C with 1 mM IPTG. The purified Rv1636 was run in 10% SDS-PAGE, and the result showed the band at the appropriate size. The protein was eluted in large concentrations with a high yield—Figure 6. The purified Rv1636 was quantified as a 0.45 mg/mL concentration using BSA as standard by Bradford assay.

### 3.8. Rv1636 Contains Prominent ATPase Activity

**Figure 7:**
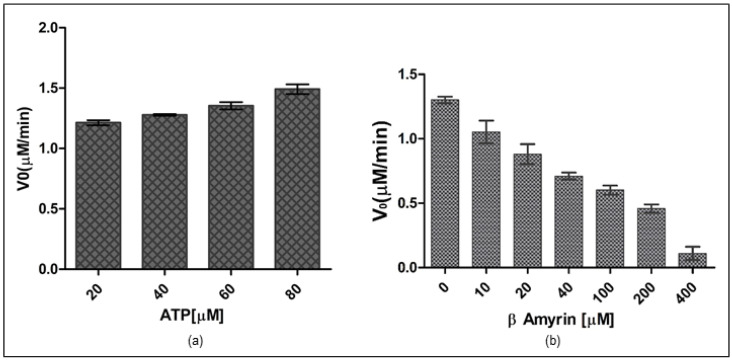
Biochemical characterization of Rv1636. (**a**) ATPase activity of Rv1636 determined by malachite green assay. The *X* and *Y*-axis show ATP concentration and enzymatic activity of Rv1636, respectively. (**b**) Effect of β-Amyrin on the ATPase activity of Rv1636. The *X* and *Y*-axis show beta-Amyrin concentration and enzymatic activity of Rv1636, respectively. The experiment was performed in triplicates, and S.D. was determined.

The malachite green test assessed the enzymatic activity, which measured the amount of inorganic phosphate produced after enzymatic ATP hydrolysis. In the presence of the ortho phosphomolybdate complex, malachite turns dark green. The reactions were carried out in a standard TMD buffer with the ingredients listed in the materials and methods section. Potassium Dihydrogen Phosphate (KH_2_PO_4_) was used to standardize the parameters. The exact concentration of Rv1636 required to act on ATP to conduct ATPase function was calculated to determine the effect of ATP on Rv1636. Different protein concentrations were utilized to determine the optimal enzyme concentration required to saturate with the ligand and establish the enzyme concentration on which further experiments would be conducted. The experiment was standardized by using various concentrations of KH_2_PO_4_ (5–100 µM) as standard. The experiment revealed that 5.0 µM Rv1636 is the optimal protein concentration for hydrolyzing ATP, with no significant increase in enzymatic hydrolysis beyond this amount. The kinetic parameters of ATP hydrolysis by Rv1636 were stated as moderate ATPase activity in the protein where *K*_m_ was 5.187 ± 0.281 µM, *V*_max_ was 1.505 ± 0.031 µM/min, *K*_cat_ was 0.053 ± 0.001 min^−1^, and *K*_cat_/*K*_m_ was 0.010 ± 0.0004 µM^−1^min^−1^. Further, on analyzing the activity in the presence of an increasing concentration of β-Amyrin, the result showed a consistent reduction in activity (see Figure 7).

### 3.9. β-Amyrin Affects the Secondary Structure Pattern of Rv1636

**Figure 8:**
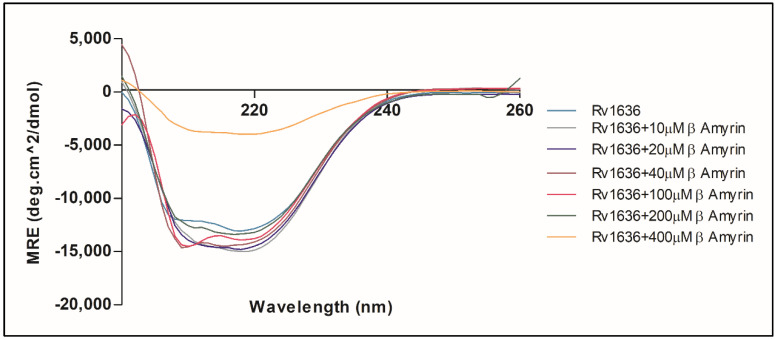
Far UV-CD spectroscopy to determine the secondary structure pattern of Rv1636 and the effect of β-Amyrin on Rv1636.

The secondary structure information of any protein is a fundamental aspect of its function and structural integrity. The secondary structure of Rv1636 was revealed by far UV-CD spectroscopy at a wavelength range from 200–260 nm. The pattern obtained by the CD spectrum in which mean residue ellipticity was plotted versus wavelength showed that the secondary structure pattern of Rv1636 was alpha-helix as it gave a narrow negative band at 208 nm and 220 nm. The result obtained by manual analysis was consistent with the data obtained by the BeStSel secondary structure prediction server and PSIPRED bioinformatic server, which also showed that most of the region of Rv1636 was an alpha helix (see Figure 8).

The effect of β-Amyrin on the secondary structure of Rv1636 was studied by mixing β-Amyrin with Rv1636 at a concentration of (10–400 µM). The secondary structure of Rv1636 was altered by the addition of 10 µM of β-Amyrin, resulting in a more negative peak at 208 nm and 220 nm. The negative peaks slightly start decreasing by adding the 10 µM of β-Amyrin to 200 µM of β-Amyrin. Adding 400 µM, β-Amyrin drastically changed the secondary structure of Rv1636 to more similar to a globular or spiral structure.

### 3.10. The Thermodynamic Interaction of β-Amyrin and Rv1636

**Figure 9:**
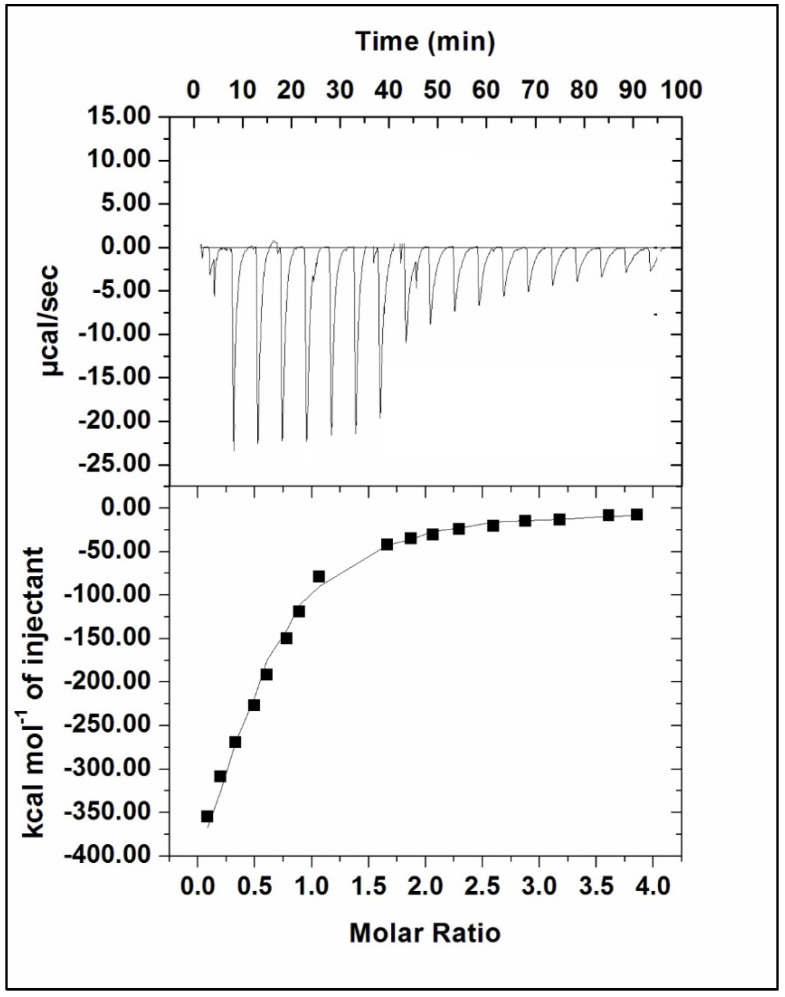
ITC isotherm of Rv1636-β-Amyrin system: The sample cell was filled with 20 µM Rv1636, while the syringe was filled with 500 µM β-Amyrin. The figure was obtained as one model binding site.

Isothermal titration calorimetry was used to describe binding energetics thoroughly, with the goal of learning more about how β-Amyrin interacts with Rv1636. It is a multidimensional method frequently used to expose the complex’s thermodynamic properties, such as the binding constant and the nature of the molecular forces involved in the binding process. The heat released or absorbed in the sample cell due to the formation or dissociation of the Rv1636_β-Amyrin complex is measured with respect to a reference cell filled with buffer (see Figure 9).

The upper panel of the Figure depicts each peak as a single injection of β-Amyrin into the Rv1636 solution. The integrated plot showing the amount of heat liberated per injection as a function of the molar ratio of drug to protein is shown in the lower panel. The binding affinity constant was on the order of 104 M−1, indicating that β-Amyrin binds strongly to Rv1636. The change in Gibb’s free energy (Δ*G*) values was negative, suggesting that the binding of β-Amyrin to Rv1636 is a spontaneous process. The difference in enthalpy (Δ*H*), entropy (Δ*S*), and other parameters obtained from isothermal titration calorimetry are given in Table 8.

## 4. Discussion

Mycobacterium tuberculosis employs different strategies for escaping from the host immune system and with respect to its drug-resistant characteristics [5,6]. As far as decade research has been concerned, a list of factors adds to bacterial persistence and increases bacterial pathogenicity. Immune modulation ability, residence in hypoxic granuloma, and pore-forming ability are features that increase bacterial pathogenicity and enhance the bacterial escaping mechanism from various medicinal drug and treatment strategies. The bacterium’s unexpected and sophisticated character necessitates a thorough understanding of every facet of host–pathogen interaction and the various mechanisms used by Mtb to escape the host immune system. The primary research goals are this examination and the genes involved in the non-replicating/persisting phase. It was also hypothesized that *Mtb* would undergo hypoxia in the granuloma after infecting the host cell. This hypoxic environment would primarily cause the bacteria’s transition from an active replicating state to a permanent non-replicating state. The probability of latent infection resurfacing in immunocompromised people is 10% every year. One-third of the world’s population is assumed to be infected with *M. tuberculosis*, with most of these people harboring latent bacilli. As a result, the latently infected form an important disease reactivation and transmission reservoir. As a result, latent tuberculosis is a significant impediment to *M. tuberculosis* control and eradication. Understanding the processes that control tuberculosis latency and reactivation could contribute to developing better TB control techniques. The present article seeks to understand crucial facts about Rv1636 which is a universal stress protein in *Mycobacterium tuberculosis* and its other species.

Rv1636 is a UspA protein which first described in *E. coli*. It is an ATP binding Usp and has a motif identified as G2 × G9 × GS. In addition to the ATP binding motif, this protein contains a conserved aspartate in the USPA domain. Furthermore, Rv1636 showed the binding affinity for cAMP, which is unusual for other known USPs. The protein–protein network analysis of the 10 proteins described earlier emphasized the Rv1636, where betweenness, closeness, radiality, and stress were the metrics that emphasize that Rv1636 is a part of the stress machinery.

The *Achyranthes aspera*, *Calotropis gigantea*, and *Calotropis procera* plants mentioned in one of our prior investigations are rich sources of β-Amyrin. The biological activity analysis revealed that β-Amyrin has a variety of pharmacological activities and is necessary for producing apoptotic events [72]. With its well-known antibacterial and anti-inflammatory properties, β-Amyrin is also a prominent candidate for antidiabetic and anti-atherosclerosis drug development.

The Rv1636 was a stable protein whose instability index was less than 40. Further, this protein was found in the cytoplasm, suggesting its involvement in the signaling system. The secondary structure prediction by the SOPMA server proved that the structure of Rv1636 was dominated by the alpha-helical pattern, in which two negative peaks can see at 208 and 222 nm. Further phylogenetic analysis showed the proximity of Rv1636 among other mycobacterial species, and the ATP binding motif was also conserved. TB 16.3 and UspA have the best interaction from the text mining approach shown in the STRING database. TB 16.3 is a conserved mycobacterial protein that plays a vital function in triggering the NF-κB signaling pathway, which is important in host protective immunity, albeit the exact mechanism is unknown. Rv2316 (UspA) is an integral membrane protein involved in the active transport of sugar across the membrane. STITCH server showed the interaction of Rv1636 with the SeMet, carboxy, chloride, nitrate, MgATP, Fe, AMPPCP, manganese, 1, 3 bisphosphate. ATP, cAMP, and hydrogen. Moreover, molecular docking was employed to predict the interaction of Rv1636 with ATP, cAMP, and beta amyrin. ATP and cAMP were used as a control to examine the docking pattern of β-Amyrin and Rv1636. The docking showed a higher binding affinity between Rv1636 and β-Amyrin as compared to ATP and cAMP. The MD simulation experiment further confirmed the interaction, which showed the significant binding between β-Amyrin and Rv1636. The lowest RMSD was found to be with the cAMP, followed by β-Amyrin and ATP, and the other parameters also declared the stable binding between β-Amyrin and Rv1636.

The Rv1636 was cloned in the pET-28a+ vector by employing EcoRI and XhoI as the restriction sites. The protein was optimally expressed at 37 °C with 1 mM IPTG. Further, the purified protein was used to determine the effect of ATP and β-Amyrin on the protein, and the experiment showed that Rv1636 contains very minute ATP hydrolyzing activity. Previous studies proved that Rv1636 has ATP binding domain and activity, but its ATP hydrolyzing activity was not reported earlier. The catalytic activity of Rv1636 was determined by a malachite green phosphatase assay that detects the released phosphate that converted into phosphomolybdate complex in the presence of ammonium molybdate compound of malachite green dye. The more phosphate release gives the greener color, detecting even the picomole concentrations of released inorganic phosphate in a solution. The result of this assay was standardized using KH_2_PO4 from ATP concentration of 0–80 µM with 5 µM Rv1636. In all experiments, the results show linear regression with an R2 value ≥ 0.95. The reactions were run in TMD buffer (50 mM Tris pH 8.0, 10 mM MgCl_2_, and 1 mM DTT), with the ultimate basal rate determined by the quantity of phosphate released in the presence of Rv1636. The malachite green dye is made out of a combination of malachite green, ammonium heptamolybdate, and Polyvinyl alcohol, which are introduced to the reaction at different times. The ammonium heptamolybdate was first added to ensure that the liberated phosphate forms the phospho-ammonium molybdate complex due to the enzyme’s hydrolyzing activity. After adding another reagent, a mixture of malachite green and PVA, this complex turns green. PVA works to keep the green hue stable throughout time. The kinetic parameters of ATP hydrolysis by Rv1636 were stated as moderate ATPase activity in the protein where *K*_m_ was 5.187 ± 0.281 µM, *V*_max_ was 1.505 ± 0.031 µM/min, *K*_cat_ was 0.053 ± 0.001 min^−1^, and *K*_cat_/*K*_m_ was 0.010 ± 0.0004 µM^−1^min^−1^. The kinetic parameters revealed that Rv1636 ATPase activity was much less than the known ATPase activity of other enzymes, e.g., hydrolysis of ATP in humans with *K*_m_ and *V*_max_ 39 µM and 2.5 nmole/mg/min respectively [a] and also with the ATPse activity of Casein Kinase [b]. Although most of the Usp contain ATP binding activity without favoring the fact that they contain the ATP binding motif or not, ATP hydrolyzing activity was not detected in the USPs yet; therefore, the clear role of ATP binding activity in USPs is still unclear. Another USP, Rv2623, was also known to interact with ABC transporter protein Rv1747, and this connection is vital for maintaining mycobacterial growth. he minute activity thus hypothesizes that Rv1636 hydrolyzes a small portion of bound ATP, which the protein can use for either transportation of small molecules or any of its other unknown metabolic activities in stress conditions. The binding of β-Amyrin to Rv1636 significantly affects the ATPase activity of the protein, and the higher concentration of β-Amyrin significantly decreased the ATPase activity to less than half. This decrease might be due to the binding pattern of both ligands to the protein. Rv1636 binds with ATP at HIS44, ASP46, ASP51, SER57, VAL60, and ALA64 and with β-Amyrin at TYR40, LEU53, VAL60, PRO83, VAL91, and ALA97. So, VAL60 is involved in both interactions, and therefore it might create a competitive environment for both ligands to bind the protein simultaneously.

The structural deformation of the Rv1636 protein in the presence of β-Amyrin, which was studied by CD spectroscopy, was another reason for the decrease in activity. By increasing the concentration of -Amyrin from 10–400 µM, the far UV-CD revealed structural alterations in Rv1636. The larger negative peaks in the β-Amyrin plot indicate that β-Amyrin increases the alpha-helical pattern; however, at a higher concentration of β-Amyrin, such as 400 µM, the structure was drastically changed and showed decreased alpha helicity. So, β-Amyrin does not keep Rv1636 in the form required for its ATPase activity, and thus β-Amyrin reduces the enzymatic activity of Rv1636.

Isothermal calorimetry was also used to confirm the interaction of β-Amyrin and Rv1636, demonstrating that Rv1636 binds to beta amyrin effectively. Following that, ligand injection into the protein was used. The protein concentration was 20 µM, and the ligand concentration was 500 µM. The combination of both partners is an exothermic reaction seen by the negative enthalpy. The binding association constant was 5.61 × 10^4^ ± 1.10 × 10^5^, which favors the binding because the binding of Rv1636 with cAMP showed a dissociation constant of 3.96 ± 0.07 × 10^6^. Because of its interference mechanism in Rv1636 activity, the interpretations suggest that β-Amyrin could be employed as a therapeutic alternative.

## 5. Conclusions

Rv1636 is a universal stress protein contributing to mycobacterial survival in different host-derived stress conditions. The protein contained ATP binding motif G2 x G9 x GS and was known to bind ATP and cAMP; however, these Rv1636 ATP and cAMP binding abilities are not interdependent. When *Mycobacterium tuberculosis* needed it, Rv1636 served as a cAMP reservoir and utilized this secondary messenger. The protein was purified using Ni-NTA resin after the gene was cloned. According to its physicochemical characteristics, this protein is cytoplasmic and stable, with an alpha-helical pattern as its predominant secondary structure. The protein interacts with cAMP, ATP, and other universal stress proteins. β-Amyrin is abundant in *Achyranthes aspera*, *Calotropis gigantea*, and *Calotropis procera* and is rich in many pharmacological properties. The protein contains very minute ATPase activity, which is affected by the addition of β-Amyrin. The involvement of VAL60 residue in both ligand interactions, maybe due to competing for binding, decreases ATPase activity. The structural deformation in Rv1636 brought on by β-Amyrin was the cause of the decline in activity. Using molecular docking and MD modeling, it was demonstrated that β-Amyrin bound to Rv1636 more strongly than ATP. Thus, the study’s findings imply that β-Amyrin may impact Rv1636’s functionality, making the bacterium more susceptible to certain stress situations.

## Figures and Tables

**Table 1:** Physiochemical profile of *Rv1636* protein.

Protein	AA	MW	pI	EC	Instability Index		Aliphatic Index	GRAVY
**Rv1636**	146	~15.31	5.51	5960	25.53	Stable	106.30	0.005

**Table 2:** Subcellular localization prediction of mycobacterial protein *Rv1636*.

S. No.	Webserver	Predicted Localization
1	TBpred AACB-based SVM/ Score	Cytoplasmic protein/3.225639
2	TBpred DCB-based SVM/ Score	Cytoplasmic protein/0.34192113
3	CELLO2GO/ Score	Cytoplasmic/4.833
4	LocTree3/ Score	Cytoplasmic/99%
5	PSORTbv.3.0/ Score	Cytoplasmic /2.50

AACB—amino acid composition based; DCB—dipeptide composition based.

**Table 3:** Secondary structure profile of *Rv1636* protein by the different webserver.

Protein Name	Webservers	Methods	Alpha Helix	Extended Strand	Random Coil
Rv1636	SOPMA	Self-optimized prediction (SOPM)	67 (45.89%)	28 (19.18%)	43 (29.45%)
	PSIPRED	Artificial neural network machine learning (NNML)	65 (44.52%)	31 (21.23%)	50 (34.25%)
	Jpred4	JNet algorithm	54 (36.98%)	34 (23.29)	58 (39.72)
	Predict Protein	Deep learning embeddings	69 (47.26%)	25 (17.12%)	52 (35.62%)

**Table 4:** Protein–protein and protein–chemical interaction studies of *Rv1636* protein.

Predicted Functional Partners	Name	Score
**Protein–Proteins Interaction (PPI)**		
TB16.3	Conserved protein tb16.3	0.864
uspA	Probable uspA, sugar-transport integral membrane protein	0.764
TB31.7	Universal stress protein family protein tb31.7	0.754
Rv1360	Probable oxidoreductase	0.748
TB9.4	conserved protein tb18.6	0.735
TB18.6	Conserved protein tb18.6	0.730
mpt63	Immunogenic protein mpt63	0.651
kdpD	Probable sensor protein kdpD	0.625
ssb	Single-strand binding protein (ssb)	0.578
ufaA1	Tuberculostearic acid methyltransferase ufaa1	0.577
**Protein–Compounds Interaction (PCI)**		
SeMet	SeMet (196.1 g/mol)	0.846
carboxy	Formic acid	0.839
chloride	Acriflavine is a topical antiseptic	0.825
nitrate	Nitric acid	0.674
MgATP	MgATP (507.2 g/mol)	0.661
Fe	Sodium nitroprusside is an inorganic compound	0.639
AMPPCP	AMPPCP (505.2 g/mol)	0.611
manganese	A trace element	0.600
1,3 bisphospho	1,3-bisphosphoglycerate	0.596
hydrogen	a hydron is the general name for a cationic form	0.596

**Table 5:** Docking hits and their binding affinities toward *Rv1636* protein.

Name of the Ligand	BE	LE	pKi	Torsional Energy
cAMP	−7.6	0.271	5.57	1.2452
ATP	−7.2	0.141	5.28	4.6695
β-Amyrin	−10.6	0.312	7.77	0.3113

LE (Ligand Efficiency (kcal/mol/non-H atom); BE (Binding Free Energy (kcal/mol).

**Table 6:** Calculated ADMET properties of the natural compound (β-Amyrin).

Absorption		Distribution	Metabolism	Excretion	Toxicity
Intestinal absorption	Water Solubility	BBB/CNS permeation	CYP2D6/ CYP1A2/ CYP2C19 inhibitor	OCT2 substrate	AMES/ROAT/Carcinogenicity/Eye irritation and corrosion
93.733%	Poor	No	No	No	No

**Table 7:** Calculated pharmacological profile of β-Amyrin (natural compound).

Pa	Pi	Biological Activity
0.977	0.001	Insulin promoter
0.976	0.002	Caspase 3 stimulant
0.944	0.001	Transcription factor stimulant
0.944	0.001	Transcription factor NF kappa B stimulant
0.939	0.004	Mucomembranous protector
0.926	0.002	Hepatoprotectant
0.923	0.004	Apoptosis agonist
0.916	0.005	Antineoplastic
0.913	0.002	Oxidoreductase inhibitor
0.909	0.002	Membrane integrity antagonist
0.903	0.002	Chemopreventive

**Pa** means probability to be active and **Pi** means probability to be inactive.

**Table 8:** Thermodynamic parameters obtained from ITC for Rv1636_ β-Amyrin system.

*K*_a_ (Association Constant) M^−1^	∆*H* (Enthalpy Change) cal/mol	∆*S* (cal/mol/deg)
*K*_a1_ = 5.61 × 10^4^ ± 1.10 × 10^5^	∆*H*_1_ = −6.58 × 10^5^ ± 1.007 × 10^6^	∆*S*_1_ *=* −2.18 × 10^3^

## Data Availability

The data that supports the findings of this study are contained within the article and supporting information.

## Supplementary Materials

The following supporting information can be downloaded at: https://www.mdpi.com/article/10.3390/biology11081214/s1. Figure S1: Structure of selected compounds for molecular docking. (a) 2D structure of Adenosine triphosphate (b) 2D structure of cyclic Adenosine monophosphate (c) 2D structure of β-Amyrin.
