# Supplementary material for: Mechanistic Insight into the Enzymatic Inhibition of β-Amyrin against Mycobacterial Rv1636: In Silico and In Vitro Approaches
Source: Biology (Basel). 2022 Aug 12;11(8):1214. doi: 10.3390/biology11081214 (PMC9405466; doi:10.3390/biology11081214)
Supplement: Supplementary file 1 [file biology-11-01214-s001.zip › biology-1801168-supplementary.pdf]

biology

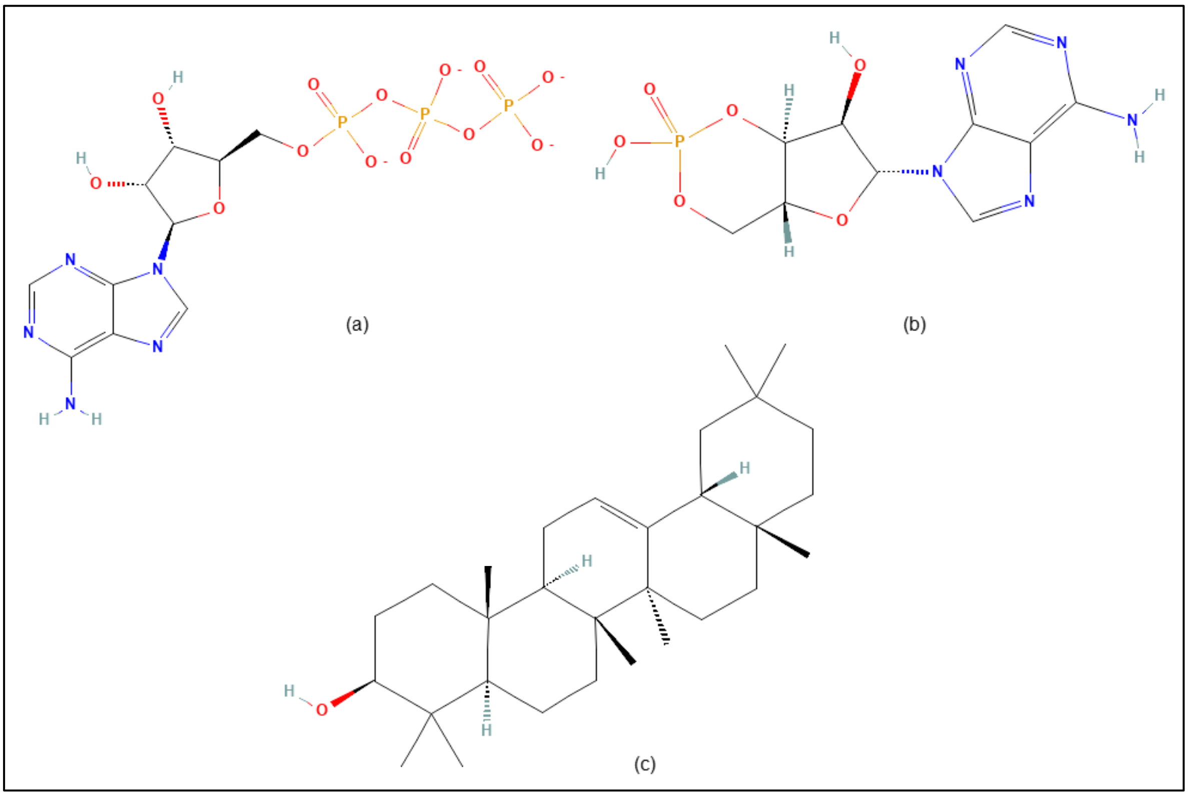

Figure S1: Structure of selected compounds for molecular docking. (a) 2D structure of Adenosine triphosphate (ATP). (b) 2D structure of cyclic Adenosine monophosphate (cAMP). (c) 2D structure of β-amyrin.
